# HIV infection results in metabolic alterations in the gut microbiota different from those induced by other diseases

**DOI:** 10.1038/srep26192

**Published:** 2016-05-18

**Authors:** Sergio Serrano-Villar, David Rojo, Mónica Martínez-Martínez, Simon Deusch, Jorge F. Vázquez-Castellanos, Talía Sainz, Mar Vera, Santiago Moreno, Vicente Estrada, María José Gosalbes, Amparo Latorre, Abelardo Margolles, Jana Seifert, Coral Barbas, Andrés Moya, Manuel Ferrer

**Affiliations:** 1 Department of Infectious Diseases, University Hospital Ramón y Cajal and Ramón y Cajal Health Research Institute (IRYCIS), Madrid, Spain; 2 Centro de Metabolómica y Bioanálisis (CEMBIO), Facultad de Farmacia, Universidad CEU San Pablo, Campus Montepríncipe, Madrid, Spain; 3 Institute of Catalysis, Consejo Superior de Investigaciones Científicas (CSIC), Madrid, Spain; 4 Institute of Animal Science, Universität Hohenheim, Stuttgart, Germany; 5 Foundation for the Promotion of Health and Biomedical Research in the Valencian Community (FISABIO) - Public Health, Valencia, Spain; 6 Network Research Center for Epidemiology and Public Health (CIBER-ESP), Madrid, Spain; 7 Department of Pediatric Infectious Diseases, University Hospital La Paz, and La Paz Research Institute (IdiPAZ), Madrid, Spain; 8 Centro Sanitario Sandoval, Madrid, Spain; 9 HIV Unit, Department of Internal Medicine, University Hospital Clínico San Carlos, Madrid, Spain; 10 Instituto Cavanilles de Biodiversidad y Biología Evolutiva (Universidad de Valencia), Valencia, Spain; 11 Department of Microbiology and Biochemistry of Dairy Products, Dairy Research Institute (IPLA), CSIC, Villaviciosa, Asturias, Spain

## Abstract

Imbalances in gut bacteria have been associated with multiple diseases. However, whether there are disease-specific changes in gut microbial metabolism remains unknown. Here, we demonstrate that human immunodeficiency virus (HIV) infection (n = 33) changes, at quantifiable levels, the metabolism of gut bacteria. These changes are different than those observed in patients with the auto-immune disease systemic lupus erythaematosus (n = 18), and *Clostridium difficile*-associated diarrhoea (n = 6). Using healthy controls as a baseline (n = 16), we demonstrate that a trend in the nature and directionality of the metabolic changes exists according to the type of the disease. The impact on the gut microbial activity, and thus the metabolite composition and metabolic flux of gut microbes, is therefore disease-dependent. Our data further provide experimental evidence that HIV infection drastically changed the microbial community, and the species responsible for the metabolism of 4 amino acids, in contrast to patients with the other two diseases and healthy controls. The identification in this present work of specific metabolic deficits in HIV-infected patients may define nutritional supplements to improve the health of these patients.

The gut microbiota should be considered as just another component of the human epigenetic landscape. Therefore, human health is a reflection of the diversity and composition of gut microbiota and its metabolic status^1^,^2^. The gut microbiota continuously reacts to perturbations such as food-intake and diseases, to preserve its homeostasis^3^. However, under some conditions, the nature of the disturbance or environmental stress is so strong that the microbiota undergo changes, acquiring a dysbiotic state^4^. Studies reveal that at least 50 pathologies are associated with gut-dysbiosis^5^. Diet- and disease-induced shifts in the composition and diversity of the total and active microbial community (assessed through 16S rDNA/RNA bar-coding) raises the question of whether the gut microbial activity may be influenced (and if so, to what extent) by these changes in community structure^6^,^7^,^8^, and if so, whether directionality exists in those changes. A second question is which diseases provoke metabolic changes that are more proximal or distant to those of healthy individuals.

Recently, changes in gut-resident microbial populations have been described in human immuno-deficiency virus (HIV) infection^9^,^10^,^11^, the metabolic consequences of which are unknown. We hypothesized that HIV-associated chronic illness, which results in major gastrointestinal tract damage^10^, would induce changes in the gut bacterial metabolism that are quantitatively different from those induced by other diseases. This was investigated by surveying the composition of metabolites produced and accumulated by gut bacteria in HIV-infected patients (n = 33). Also included in this study were cohorts of individuals with the prototypical auto-immune disease, systemic lupus erythaematosus (SLE; n = 18)^12^ and individuals with infectious diarrhoea caused by toxigenic *Clostridium difficile* (CDADt^+^; n = 6)^8^, used here as study cases. Microbial metabolites have been reported as the best indicators of disturbances in the gut metabolism^8^,^12^. To capture results for a wide spectrum of HIV immune-pathogenesis, we recruited and investigated viremic untreated HIV-infected individuals (VU; n = 11), immunological antiretroviral therapy (ART) responders (IR; n = 14) and non-responders (INRs; n = 8) (IRs and INRs, ≥350 and <350 CD4+ T-cell counts/uL after >2 years of viral suppression, respectively). The control group was composed of healthy individuals (HCs) (n = 16). To reduce the possibility that the characterization of the HC group would be affected by factors known to influence the gut microbial metabolism, such as age, diet and medication, healthy individuals with similar characteristics were selected. Body mass index (BMI) was the only differential factor: individuals (n = 7) with a BMI ≤24.83 kg/m^2^ were considered to be ‘healthy lean’ (HCl), while those (n = 9) with a BMI ≥25.24 kg/m^2^ formed the obese or so-called ‘high BMI’ healthy controls (HCh)^8^.

We found that HIV infection leads to a significant and distinct impact on the metabolism of the gut ecosystem compared with the other conditions studied. The results suggest that the metabolite fluxes associated to gut microbe’ activity are disease-dependent, which may influence distinct health risks.

## Results

### Chronic HIV infection alters the composition of gut bacterial metabolites: comparison with other diseases

Metabolites of gut bacteria from the 73 individuals were analysed by liquid chromatography couple with electrospray ionization quadruple time-of-flight mass spectrometry (LC-ESI-QTOF-MS) in both negative and positive modes. Note that we are aware that HIV associates with gastrointestinal tract damage and thus human host cells may also coexist with bacterial cells in the gut environment. To avoid the influence caused by host cells, we used a bacterial enrichment separation protocol that was proven to be efficient to eliminate eukaryotic cell contamination as revealed by the analysis of 18S rRNA genes^8^. A full description of the methods is available in the Methods section. The objective of this study was to identify whether HIV induced metabolic alterations in the gut ecosystem, and if so, whether such alterations were different from those induced by other diseases and compared with healthy controls.

**Figure 1:**
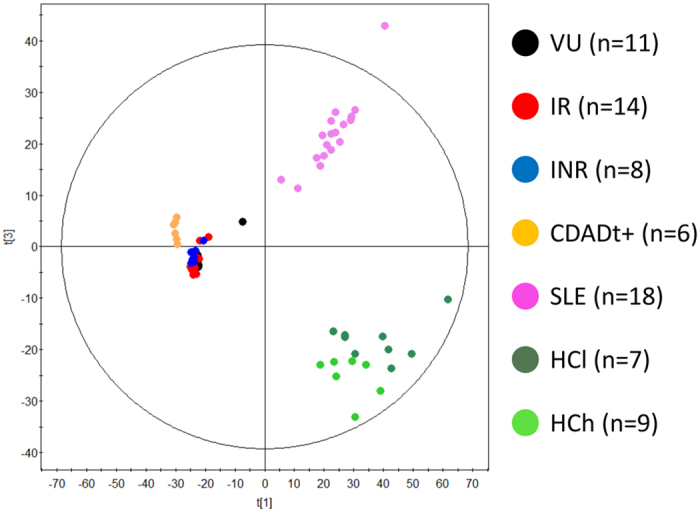
Composition of metabolite profiles inside gut microbial cells that were previously separated from stool material. A PCA plot shows the models that were built with the raw data (LC-MS (−)) that satisficed the quality assurance criteria proposed by Godzien *et al.*^13^ (variables that were present in at least 50% of the samples of each group and that were present in at least 80% of the QCs that obtained a coefficient of variation less than 30% or present in less than 20% of the QCs). The following codes were established: HCh for healthy controls with a BMI higher than 25.24 kg/m^2^; HCl for healthy controls with a BMI lower than 24.83 kg/m^2^; SLE for patients with systemic lupus erythaematosus; CDADt^+^ for patients with *C. difficile* producing toxins; VU for untreated HIV-infected individuals; IR for immunological ART responders who were HIV-infected individuals; and INR for immunological ART non-responders who were HIV-infected individuals. The robustness of the sample clustering has three main contributors. First, the PCA is a non-supervised model, which means that the distribution of the samples that was plotted is due only to the directions of the principal components that maximise the explained percentage of variance (R^2^). Second, raw data are plotted, including all possible sources of variability in addition to the biological sources. Third, the robustness of the analytical procedure was demonstrated by the tight clustering of the QCs in the non-supervised PCA models, showing that separation among the groups was due to real biological variability and not to analytical variance (random). For a full description of the HCh, HCl, SLE, and CDADt^+^ samples and patients, see recent publications^8^,^12^. Similar separation of the groups was observed also in LC (+), and this is why only the datasets for LC (−) are shown. Note that our previous investigations revealed that the segregation of samples is highly similar regardless of the separation technique used to obtain the metabolomics fingerprint; this technique clearly influences only the part of the metabolome than can be seen and does not introduce biases in the segregation process.

Out of 357,941 mass features, 24,435 passed the filtering criteria proposed by Godzien *et al.*^13^ A scatter plot based on principal component analysis (PCA) scores, obtained from the set of metabolites that fulfilled the filtering criteria for selection, revealed a clear separation between healthy individuals sub-categorized as either low BMI (HCl) or high BMI (HCh) (Fig. 1), as previously reported^12^. This separation was not observed in patients experiencing any of the three diseases investigated. This is in agreement with previous study suggesting that in healthy subjects in whom no stronger pressure than BMI exists, BMI becomes a driving factor determining microbes´metabolic activity^12^. By contrast, the absence of high-body-weight and lean sub-groups in patients demonstrates that the presence of a disease seems to be a stronger pressure of bacterial metabolism than BMI. It also revealed that both HC sub-groups were distinct from patients with immune (SLE) or viral (HIV) diseases or infectious diarrhoea (CDADt^+^) (Fig. 1). The metabolic changes induced in the gut ecosystem by the viral agent (HIV) and by *C. difficile* were sharper (Fig. 1). This suggests a more specific cascade of functional consequences, explanations for which include the following factors: *i*) the numerous structural (enterocyte apoptosis and the loss of epithelial integrity), functional (errors in the production of mucin/IgA), and immunological (loss of sub-mucosal lymphocytes) defects secondary to HIV infection^10^,^14^; and *ii*) the presence of inflamed intestinal lining due to abnormalities in mucin production secondary to antibacterial therapy to treat *C. difficile*^15^. SLE and obesity (in HCh) are pathologies that exhibited more heterogeneous profiles (Fig. 1), most likely because the precipitating agents are also heterogeneous and because, to our knowledge, obesity^16^,^17^ and SLE^18^ do not have such dramatic effects on the architecture of the gut mucosa.

**Figure 2:**
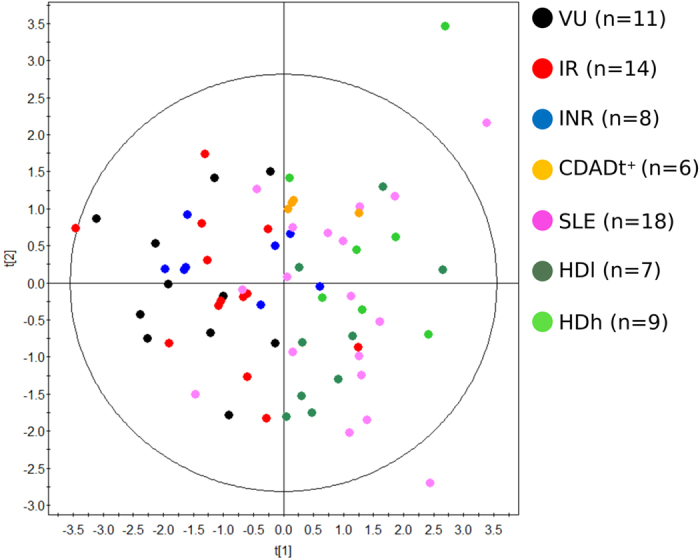
PCA score plots for multivariate statistical analysis. Clinical variables examined include residential zone, age, gender, body mass index, glucose, creatinine, triglyceride, cholesterol, CD4+ T-cells, Nadir CD4+ T-cells, CD4/CD8 ratio and % CD8+ HLA-DR + CD38+ T cells levels, the type of medication and sexual preferences (Supplementary Table S1). The variables between two of the different principal components are: R^2^ = 0.541; Q^2^ = −0.093.

The scatter plot in Fig. 1 suggests that diseases introduced the major variation to the microbes’ metabolic activity. To determine whether other factors such as residential zone, subject characteristics such as age, gender, and body mass index, clinical variables such as glucose, creatinine, triglyceride and cholesterol levels, and adaptive immune markers such as CD4+ T-cells, Nadir CD4+ T-cells, CD4/CD8 ratio and % CD8+ HLA-DR+CD38+ T cells, the type of medication and sexual preferences (Supplementary Table S1), are also involved in alterations in the gut microbiota, a non-supervised multivariate statistical analysis (PCA model) was performed. As shown in Fig. 2, as far as no clear group separation can be observed it is concluded that the samples cluster based on the impact of the diseases on the gut metabolism (Fig. 1) and not due to other sampling factors (Fig. 2).

**Figure 3:**
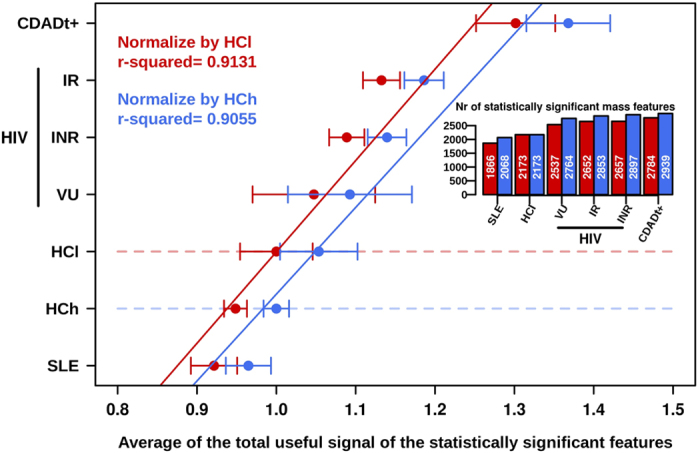
Dysbiosis ranking correlation plot based on the impact of the diseases on the gut microbial metabolome. Quantifications based on the number of metabolites (shown in the inset) and the total useful signals (X axe) that were statistically (*p* < 0.05) altered in each disease (Y axe, arbitrary) versus HCl (in red) and HCh (in blue) are shown. Values represent the median ± range.

The scatter plot in Fig. 1 also suggests that the changes induced by HIV infection are different from those caused by the other diseases. As the PCA model provide a non-supervised multivariate statistical analysis (*p* < 0.05), the separation should be considered significant. Following on from this, we further analysed whether directionality exist in the changes in bacterial activity. Also we evaluate which diseases are accompanied by a loss or increase in metabolic activities, and to what extent as compared to a healthy individual. As it may happened that the extent of changes may be influence by the reference group, we decided to use both the lean and obese healthy individuals as control groups. For that, the changes in gut microbiota activity were first analysed by quantifying the number of metabolites that differed (*p* < 0.05; see Supplementary Table S2) between each group and the lean healthy controls (baseline for comparison). These changes were used as an indicator of the level of metabolic alterations in the gut ecosystem. We found that, compared to lean controls, HCh and SLE elicited fewer numbers of metabolic alterations than CDADt^+^ and HIV (Fig. 3 inset). The measurement of the total peak area of the differentially observed metabolite features was also used as a parameter to measure changes in metabolite flux. We found that high BMI (in HCh) and SLE negatively impacted metabolite flux compared with HCl, with the total peak area of metabolites produced or accumulated by gut bacteria decreasing by 5% and 8%, respectively (Fig. 3). In contrast, CDADt^+^ increased metabolite flux by approximately 30% compared with HCl (Fig. 3). HIV also increased metabolite flux, which was observed in the following order: IR (13% increase) >INR (9% increase) >VU (5% increase), when the groups were arranged from highest to lowest (Fig. 3). When the reference group was the HCh, similar results were obtained (Fig. 3), implying that the directionality of the changes remains independently of the reference group. Only small differences were observed within the numbers of metabolic alterations between each group and the control group (Fig. 3 inset), most likely due to the fact that the obese individuals possess a different spectrum of metabolites.

### Metabolic deficits in HIV-infected patients: amino acid metabolism

We observed that 332 mass features were detected at quantifiable levels only in the gut microbiota of patients with both SLE and CDADt, whereas 139 accumulated only in the gut microbiota of HIV-infected patients (see Supplementary Table S2). These features can be considered to be metabolic biomarkers that distinguish HIV from SLE and CDADt^+^. Empirical formulas and identities were assigned and confirmed for these masses using LC-MS (see Supplementary Table S2), and the biological significance was revealed.

Among the major significant changes observed was that HIV infection, but not SLE or CDADt^+^, associated with an impaired metabolic capacity of the gut microbiota to produce the following three amino acids: proline (Pro), phenylalanine (Phe) and lysine (Lys). The accumulated levels of these amino acids inside the gut bacteria were below the detection limit in all three groups of HIV-infected patients, but were significantly accumulated in patients with both SLE and CDADt^+^ and to a higher level in healthy controls (*p* < 0.00026; Table 1). These results agree with the finding that intermediates of Pro, Phe and Lys biosynthesis and degradation were also present at unquantifiable levels in HIV-patients (Table 1; Supplementary Table S2; *p* < 0.005). Their significant depletion in the gut microbiota of HIV-infected patients appears to agree with the fact that the expression level of proteins participating in ‘Pro, Phe and Lys metabolism’ were below the detection limit in the gut microbiota of HIV-infected patients. This was found by measuring the protein expression profiles in the gut bacterial samples of 16 out of 33 (VU: 5; IR: 7; INR: 4) HIV-infected patients (see raw data in the Supplementary Table S3) using a shotgun meta-proteomic approach (see Methods section).

In contrast, 3-hydroxyanthranilate, a product of the kynurenine pathway during tryptophan catabolism, accumulated in the gut bacteria of all three groups of HIV-infected patients but was below the limit of detection in SLE and CDADt^+^ patients and healthy controls (*p* < 0.00016; Table 1). These results are in agreement with previous observations suggesting a relationship between a ‘HIV-associated microbial community’ and a higher capacity to catabolize tryptophan via the kynurenine pathway in the dendritic cells and macrophages of the mucosa^9^.

## Discussion

As the intestinal microbiota has a direct contact with the gastrointestinal tract mucosa, it is anticipated that its alteration might culminate in events that influence human health^19^. Compositional abnormalities have been shown to be the consequence of diseases such as HIV^9^,^10^,^11^ and *C. difficile* infections^15^ that directly induce extensive gastrointestinal tract damage. However, modifications have also been reported for other diseases that do not have an obvious impact in the colonic mucosa, such as the SLE immune disease^18^. It would be expected that infections resulting in greater gastrointestinal tract damage would more profoundly alter the microbial activity in the gastrointestinal tract. However, no such experimental evidence has been reported. To investigate this, cohorts of individuals with HIV-associated chronic illness, the auto-immune disease SLE and *C. difficil*e-associated diarrhoea were used as study cases. The baseline for comparisons was healthy controls, 9 of whom were characterized by high BMI values. By measuring differences in metabolites accumulated in gut bacteria as an indicator of metabolic alterations, we investigated whether and to what extend these diseases/disorders alter gut microbial metabolism^12^.

The close clustering of all disease-associated microbial metabolites (including HIV infection), with each disease exerting different levels of modifications, demonstrates that diseases are the dominant factor regulating the gut microbial activity, regardless of environmental or individual (including BMI) characteristics. This is not the case in healthy subjects, where the absence of strong selection pressures allows individual characteristics, such as the BMI, to influence the gut microbial activity^12^. We further demonstrate that a trend in the nature (Fig. 1) and directionality (Fig. 3) of the changes exists in the gut bacteria metabolism, according to the type of disease (HIV infection, SLE and *C. difficil*e-associated diarrhoea) or disorder (high BMI). Indeed, compared with healthy lean individuals, high BMI (obesity) and SLE most likely provoke fewer alterations and an overall lowering of metabolic flux in the gut environment. In contrast, HIV (viral) and *C. difficile* infections provoke major alterations and an increment in the distribution of metabolite-wide flux, in agreement with the fact that both infections induce major structural changes in the intestines. This is a new and important finding that is demonstrated here for first time. This finding may be particularly significant in opening new research avenues to study the impact of different diseases on the gut microbial activity. Previously, variations in gut microbial activity have been shown to be associated with the production and circulating levels of metabolites that directly influence host health^20^.

We demonstrate that the microbe’ metabolic activity is drastically changed by the viral infection or, most likely, by the numerous structural, functional and immunological changes in the gut of HIV infected patients^10^. Interestingly, our data also revealed that the effect of HIV infection on gut bacterial activity was accentuated among immunological ART responders with an adequate CD4+ T cell recovery, as revealed by the higher number of alterations (Fig. 3) in those patients compared with viremic untreated and immunological-non responders. Those changes in the context of immune recovery deserve further analysis and suggest that gut microbiota might influence the extent of immunological recovery under ART. Furthermore, our data demonstrate that the viral infection, as compared to other diseases, drastically changed the microbial community and the species responsible for the metabolism of a set of amino acids are significantly decreased. Thus, our results demonstrate that HIV-induced gut inflammation is associated with a dysbiotic gut ecosystem that is unable to metabolize a specific group of amino acids composed of Pro, Phe and Lys. These amino acids, in contrast, are metabolized by the bacteria of individuals with SLE and *C. difficile* infection and in healthy controls. This may have long-term consequences resulting in nutritional deficits, including the wasting syndrome observed in patients with advanced HIV disease. This information may help in designing, for example, nutritional supplements to enhance gastrointestinal tract physiology^21^.

In contrast, the accumulation of metabolites produced during tryptophan catabolism is characteristic of HIV infected patients, compared with SLE and CDADt^+^ patients and healthy controls. Emerging data suggest that the excess of tryptophan degradation in HIV-infected patients results from the induction of IDO-1 (present in dendritic cells and macrophages of the mucosa) by a local pro-inflammatory environment or possibly as a result of the presence of some bacteria^9^,^11^. There is increasing awareness that the excess kynurenine might negatively affect different processes that are key to mucosal immunity^9^, and induction of this pathway is being repeatedly linked with adverse clinical outcomes^22^,^23^,^24^,^25^,^26^. It is therefore plausible that the human enzyme responsible for the degradation of tryptophan is induced in HIV patients by components of the gut microbiota. In addition, gut bacteria might contribute to the amount of kynurenine in the mucosa and the gut environment by assimilating and accumulating it (and its degradation products) inside bacterial cells. Thus, gut bacteria might impact the availability of kynurenine in the gastrointestinal tract, thereby decreasing its effects in mucosal and systemic immunity^9^. Interestingly, kynurenine degradation products accumulated at higher levels among immunological ART responders (Table 1). As the kynurenine pathway is at the crossroads of metabolism and immunity and plays a crucial role in the establishment of inflammation while also playing an opposing role in the control of acute and chronic infections^26^,^27^, these data indicate that bacterial communities are key players in the maintenance of not only chronic inflammation in chronic HIV infection but also possibly in immune recovery.

**Figure 4:**
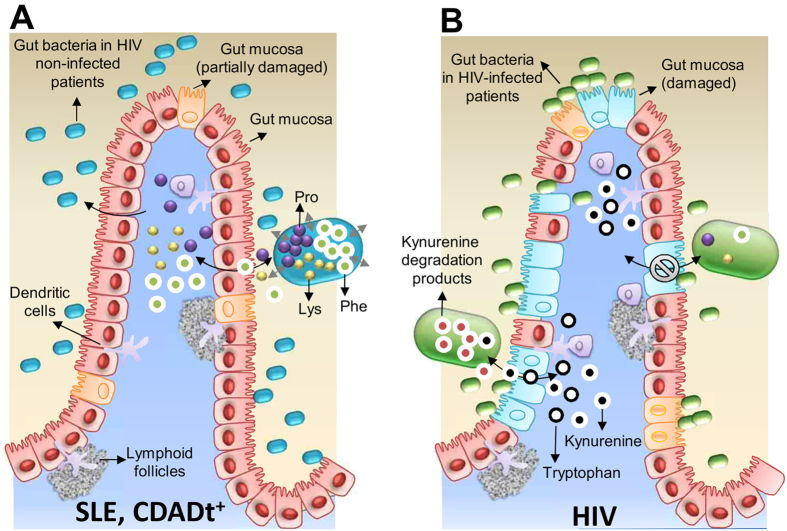
HIV infection results in metabolic alterations in the gut microbiota that differ from those induced by other diseases. **(A)** The intestinal epithelium in patients with SLE and CDADt^+^. The gut microbiota has the capacity to metabolize Pro, Phe and Lys, amino acids that accumulate inside bacterial cells. These amino acids are excreted by the bacteria into the gastrointestinal tract environment and are transported through the mucosa. The metabolism of tryptophan by gut bacteria is not affected. **(B)** The intestinal epithelium in an HIV-infected individual. These individuals are characterized by a gut microbiota comprising a distinct set of bacterial species that has a reduced capacity to synthesize Pro, Phe and Lys. Therefore, their ability to transport these amino acids to the human cells is also impaired. In contrast, tryptophan and kynurenine produced in the dendritic cells and macrophages of the mucosa can be transported to the gut environment and can be metabolized by gut bacteria.

In summary, this study provides the first direct experimental evidence that HIV-associated chronic illness alters gut microbial activity and that the alterations are different than those induced by other diseases (summary scheme in Fig. 4). The results of this study reinforce the hypothesis that alterations in metabolite composition and in the richness of the gut bacterial metabolites could partially be reflecting disease-dependent gastrointestinal tract damage. This study identifies an urgent need in gut microbiota research and clinical studies to measure disease-specific metabolic alterations.

We are aware that a substantial fraction of the metabolites in the metabolome remain unknown. However, the identified metabolites are assumed to represent the dominant and active metabolic pathways in each gut ecosystem. We cannot be sure that the absence (or a level below the detection limit) of a metabolite in a sample implies that it is fully absent *in vivo*, but it does imply that it represents a minor pathway. In addition, we would like to stress that one limitation of our study is that we only investigated 3 diseases that are quite different in nature and aetiology. We found disease-specific influences upon gut microbial activity. We believe that this study constitutes a proof of concept, as we found, for the first time, disease-specific influences upon gut microbial activity. Therefore, extending the study to other diseases may be an important research opportunity. Finally, although this study showed metabolic associations, it did not demonstrate causality, which certainly deserves further investigation.

## Methods

### Study design, participants, setting, eligibility and methods

We recruited 33 HIV-infected individuals (VU: 11; IR: 14; INR: 8). HIV-infected individuals on ART were representative of a middle-aged long-term treated population without metabolic abnormalities. Subjects were recruited from the HIV clinics of two University hospitals in Madrid, Spain (University Hospital Clínico San Carlos and University Hospital Ramón y Cajal). The inclusion criteria were serologically documented HIV infection and an age of 18 years old or older. The following exclusion criteria were used: concomitant medications, the use of systemic antibiotics during the previous three months, and any acute or chronic condition other than chronic HIV infection, including gastrointestinal symptoms (constipation, bloating or diarrhoea) or co-infections by hepatitis B or C viruses. SLE patients (n = 18) were recruited in accordance with approved guidelines and regulations from the updated Asturian Register of Lupus and fulfilled at least four of the American College of Rheumatology criteria for SLE^12^. Patients (n = 6) who carried toxin-producing *C. difficile* were recruited at the Hospital Clínico Universitario in Valencia (Spain)^8^. Controls (n = 16) were healthy volunteers from the same population as for SLE patients^12^. Full descriptions of SLE, and CDADt^+^, and healthy individuals are published elsewhere^8^,^12^.

Supplementary Table S1 summarizes the characteristics of the individuals included in each of the groups that were investigated.

### Ethics statement

This study conformed to the principles or the Declaration of Helsinki and Good Clinical Practice Guidelines and was approved by the Independent Ethics Committees of recruiting institutions. For HIV-infected individuals: University Hospital Clínico San Carlos [approval number 11/284], ceic.hcsc@salud.madrid.org, and University Hospital Ramón y Cajal, ceic.hrc@salud.madrid.org. For SLE and healthy individuals: CSIC and the Regional Ethics Committee for Clinical Research (Servicio de Salud del Principado de Asturias) [approval number AGL2010-14952; grant title “Towards better understanding of gut microbiota functionality in some immune disorders”]. For CDADt^+^ patients: Hospital Clínico Universitario in Valencia (Spain) [approval number 2012/196; grant title “Estudio explanatorio sobre el impacto de la comunidad bacteriana intestinal en el desarrollo de la colitis asociada a *Clostridium difficile*”]. Informed consent was obtained from all subjects.

### Metabolite measurements in gut bacteria samples

Fresh stool samples were collected from each subject, frozen immediately and stored until processing at −80 °C. After thawing, the gut bacteria were separated from faecal material, and the metabolites that had accumulated intracellularly were extracted from equal amounts of microbial cells per sample by adapting a previously reported method in which a two-step extraction method produces the optimal extraction efficiency for both polar and hydrophobic metabolites^8^,^12^. Briefly, microbial cells were separated from the faecal matrix by mixing 0.4 g of faecal sample with 1.2 mL of phosphate-buffered saline (PBS) (1:3 *w*/v faeces to PBS ratio). Following re-suspension (by 1 min of vigorous vortexing), the samples were then centrifuged at 1,000 *g* at 4 °C for 1 min to remove faecal debris. The supernatant (1.2 mL) was transferred to a 2-mL Eppendorf tube and centrifuged at 13,000 *g* at 4 °C for 5 min to pellet the cells. This protocol was repeated 3 times. The cell counts in the bacterial pellets were then immediately analysed, as previously described^28^, so that the same amount of bacterial cells was used in each extraction experiment. A total of 10^8^ gut bacterial cells per sample were used for methanol extractions, in which 1.2 mL of cold (−80 °C) high performance liquid chromatography (HPLC)-grade methanol was added. The samples were then vortex-mixed (for 10 s) and sonicated for 30 s (in a Sonicator® 3000; Misonix) at 15 W in an ice cooler (−20 °C). This protocol was repeated twice with a 5-min storage period at −20 °C between each cycle, and the final pellet was removed following centrifugation at 12,000 *g* for 10 min at 4 °C. Immediately after the methanol extracts were obtained, the methanol solution was stored at −80 °C, and the remaining cell pellet was re-suspended in 1.2 mL of cold (4 °C) HPLC-grade H_2_O and subjected to 3 cycles of sonication for 20 s (in a Sonicator® 3000; Misonix) at 15 W in ice water. The samples were incubated on ice for 2 min between cycles. The final pellet was removed following centrifugation at 12,000 *g* for 10 min at 4 °C. Immediately after the H_2_O and methanol extracts were obtained, a mixture was prepared by combining equal amounts (1 mL) of each of the extracts. Once prepared, the final solution was stored in 20-mL penicillin vials at −80 °C until they were analysed. Prior to analysis, a pre-processing step was performed to eliminate impurities and particles by mixing the samples with (HPLC)-grade acetonitrile (1:1) and subsequently centrifuging them (13,000 *g* at 4 °C, 10 min). Then, all supernatants were immediately analysed simultaneously by liquid chromatography couple with electrospray ionization quadruple time-of-flight mass spectrometry (LC-ESI-QTOF-MS; LC: 1290 infinity, Agilent, QTOF-MS: Agilent 6550 iFunnel) in positive and negative modes. The full details for chemical and reagents, methods, and preparation of quality controls (QCs) are available elsewhere^8^,^12^.

### Metabolite data treatment, statistical analysis and identification

For metabolomic data treatments, the Molecular Feature Extraction tool in the Mass Hunter Qualitative Analysis software (B.06.00, Agilent) was used. The alignment of the raw data was performed using Mass Profiler Professional software (version 13.0, Agilent). The variables were then filtered according to the filtering criteria proposed by Godzien *et al.*^13^: variables that were present in at least 50% of the samples of each group and i) present in at least 80% of the QCs that obtained a coefficient of variation less than 30% or ii) present in less than 20% of the QCs. Models were subsequently built using SIMCA-P+ software (12.0.1.0, Umetrics). The full details for data treatment and statistical analysis can be seen in recent publications^8^,^12^. Mann-Whitney *U* tests were used to perform all of the comparisons, followed by Benjamini-Hochberg *post hoc* correction (*p* < 0.05). The resulting list of accurate masses that significantly differed between groups was searched using the CEU Mass Mediator search tool (http://biolab.uspceu.com/mediator; error ±5 Da) to obtain tentative identifications. This procedure was performed independently for each analytical platform. The identity of the amino acids selected according to their significance in the class separation was further confirmed using LC-MS/MS in the same LC-ESI-QTOF-MS. Ions were targeted for collision-induced dissociation (CID) fragmentation based on the previously determined accurate masses and retention times. Their identity was confirmed by comparing the fragments that were obtained with the structure of the proposed compound in the MS/MS spectra in a public database (METLIN: https://metlin.scripps.edu/metabolites_list.php) or against commercially available standards.

### Protein extraction and mass spectrometry and data analysis

Protein extracts were isolated as previously described^29^, with small modifications. Briefly, equal amounts of enriched gut bacteria samples (10^8^ cell in total per sample), obtained as described above, were re-suspended in 1.2 mL of BugBuster^®^ Protein Extraction Reagent (Novagen, Darmstadt, Germany) for 30 min with shaking (250 rpm). Then, the samples were disrupted by sonication using a pin Sonicator^®^ 3000 (Misonix, New Highway Farmingdale, NY, USA) for a total time of 2 min (10 watts) on ice (4 cycles × 0.5 min with 1.0 min ice-cooling between each cycle). The extracts were then centrifuged for 10 min at 12,000 *g* and 4 °C to separate cellular debris and intact cells, and the supernatants were then carefully aspirated (to avoid disturbing the pellet), transferred to new tubes, and stored at −80 °C until use.

Protein extracts were thawed on ice, and 50 μg of each sample was precipitated using 1 mL 20% trifluoroacetic acid at 4 °C for 40 min followed by 15 min of centrifugation at 12,500 *g* and 4 °C. Protein pellets were washed twice in ice-cold acetone and dried using vacuum centrifugation at 30 °C. Prior to 1D-SDS-PAGE (12%, 60 min at 20 mA), pellets were solubilized in 15 μL Laemmli-buffer by 5 min of sonication followed by vortexing. Samples were incubated for 8 min at 95 °C to reduce disulphide bonds, and 12 μL of each sample was run on a gel. Proteins were separated by less than 1 cm, cut, and subjected to in-gel trypsin digestion overnight at 37 °C^30^. The obtained peptides were purified and desalted using C_18_ StageTips^31^,^32^, dried using vacuum centrifugation, and prior to LC-MS/MS measurements, they were reconstituted in 20 μL 0.1% trifluoroacetic acid/2% acetonitrile.

Each sample of 8 μL was injected using an autosampler and concentrated on a trapping column (Pepmap100, C_18_, 100 μm × 2 cm, 5 μm, Thermo Fisher Scientific) with water containing 0.1% formic acid and 2% acetonitrile at flow rates of 4 μL min^−1^. After 10 min, the peptides were eluted into a separation column (PepmapRSLC, C_18_, 75 μm × 50 cm, 2 μm, Thermo Fisher Scientific). Chromatography was performed using 0.1% formic acid in solvent A (100% water) and B (100% acetonitrile). The solvent B gradient was set from 4 to 8% for the first 15 min and subsequently increased to 20% for the next 110 min. After this, solvent B was increased from 20% to 30% over 15 min, from 30% to 40% over 10 min, and finally switched to 90% solvent B for an additional 10 min using a nano-high pressure liquid chromatography system (Ultimate 3000 UHPLC, Thermo Fisher Scientific). Ionized peptides were measured and fragmented using a Q Exactive mass spectrometer (Thermo Fisher Scientific). For an unbiased analysis, continuous scanning of the eluted peptide ions was performed between 400–1200 *m*/*z* and then automatically switched to MS/MS higher energy collisional dissociation mode with twelve MS/MS events per survey scan. For MS/MS HCD measurements, a dynamic precursor exclusion of 30 s per peptide match and an apex trigger of 2 to 30 s were enabled.

To achieve a more precise evaluation of protein abundances and to decrease the false discovery rate of peptide identifications, a two-step database search was used^33^,^34^. Raw MS and MS/MS data were first processed using Thermo Proteome Discoverer software (v.1.4.1.14) and Mascot Server (v. 2.4.1) and then independently searched against the NCBInr databases (v. April 25, 2015) for bacteria. Oxidation of methionine was set as the variable modification, and carbamidomethylation of cysteine was set as the fixed modification. Precursor ion tolerance was defined at 10 ppm, and fragment ion tolerance was set to 0.02 Da. Furthermore, all peaks except for the top 12 peaks per 100 Da in each MS/MS were removed to remove noise from the spectra before identification. To extract a more specific protein Fasta database, the default settings of Thermo Proteome Discoverer were kept, including protein grouping with a minimum peptide confidence set to “medium” and a delta Cn of 0.15. A strict maximum parsimony principle was used.

At the second step, the obtained in-house database was used for label-free protein quantification by applying the LFQ modality of the MaxQuant software (v. 1.5.3.8). Cysteine carbamidomethylation was set as the fixed modification, and methionine oxidation was set as the variable modification. Re-quantification was enabled. Two missed cleavage sites were allowed for protease digestion, and peptides were required to be fully tryptic. Other parameters of the software were kept at the default settings. These included a peptide and protein FDR below 1%, at least 1 peptide per protein, a precursor mass tolerance of 4.5 ppm and a fragment ion mass tolerance of 20 ppm. The LFQ abundance values were subjected to statistical analyses using Primer 6 software (v. 6.1.16). Significant differences between treatment groups were determined by performing one-way Analysis of Similarities (ANOSIM) and Similarity Percentages (SIMPER) analyses.

## Additional Information

**How to cite this article**: Serrano-Villar, S. *et al.* HIV infection results in metabolic alterations in the gut microbiota different from those induced by other diseases. *Sci. Rep.* **6**, 26192; doi: 10.1038/srep26192 (2016).

## Supplementary Material

Supplementary Information

Supplementary Table S1

Supplementary Table S2

Supplementary Table S3

## Figures and Tables

**Table 1:** The impact of HIV, SLE and CDAD on the amino acid metabolism of gut bacteria.

Metabolite	Mass (*m*/*z*)	Peak area (arbitrary units)						*p* value
		SLE	CDADt^+^	VU	IR	INR	HC^5^	
Proline	115.0628 (LC+)	1,029,779 (133,679–1,790,834)	184,318 (122,879–345,450)	0^1^,^4^	0^1^,^4^	0^1^,^4^	1,259,095 (86,950–1,944,313)	<0.00026
Phenyl alanine	165.0796 (LC+)	476,712 (71,275–2,687,654)	644,264 (607,695–702,184)	0^2^,^4^	0^1^,^4^	0^1^,^4^	1,573,890 (201,340–4,241,643)	<0.00026
Lysine	146.1054 (LC-)	23,299 (9,153–36,369)	9,929 (6,619–18,407)	0^3^,^4^	0^1^,^4^	0^1^,^4^	36,045 (12,548–62,984)	<0.00021
3-Hydroxy-anthranilate	153.0428 (LC-)	0	0	4,187 (4,187–13,706)	13,973 (6,991–16,557)	5339 (3,559–15,461)	0	<0.00016

The Table shows the median ± interquartile range of key metabolite features that were differentially (and statistically) accumulated in the HIV (VU, IR and INR), SLE and CDADt^+^ groups of patients and healthy controls (*p* values < 0.05; for exact values see Supplementary Table S2). Separation and quantification were performed as described in the Methods section.

^1^Abundance levels of ornithine (*m*/*z* 132.0899) and feruloyl putrescine (*m*/*z* 264.1467) involved in the biosynthesis and degradation of proline, respectively, was below detection limit.

^2^Abundance level of 6-deoxy-5-keto-fructose-1-phosphate (*m*/*z* 242.0188) and chorismate/prephenate (*m*/*z* 226.0478) involved in the biosynthesis of phenylalanine, and the further degradation product cinnamate (*m*/*z* 148.0525) were below detection limit.

^3^Abundance levels of homoserine (*m*/*z* 119.0583) and N-acetyl-2-amino-6-oxopimelate (*m*/*z 231.0732*) involved in the biosynthesis of lysine, and the further degradation product N-acetyl-lysine (*m*/*z* 188.1159) were below detection limit.

^4^At least 34 di- and tripeptides containing Pro, Phe and/or Lys (*m*/*z* 186.0997, 228.1467, 257.1383, 259.189, 259.1902, 260.1375, 262.079, 273.1801, 278.1626, 337.1145, 348.1643, 364.1753, 371.2153, 373.208, 373.2214, 384.2497, 386.1753, 386.1915, 388.233, 398.1946, 398.1972, 400.2276, 403.2204, 409.1673, 414.1706, 417.2011, 432.2021, 432.2029, 442.2119, 445.2682, 450.2228, 468.2175, 475.1798, and 537.2395), were also below the detection limit in HIV-infected patients. Thus, a lower ability of gut ecosystem to transport and convert peptides to free Pro, Phe and Lys amino acids could be suggested.

^5^HC included both lean and high BMI individuals.
