# Supplementary material for: HIV infection results in metabolic alterations in the gut microbiota different from those induced by other diseases
Source: Sci Rep. 2016 May 18;6:26192. doi: 10.1038/srep26192 (PMC4870624; doi:10.1038/srep26192)
Supplement: Supplementary Information [file srep26192-s1.doc]

# HIV infection results in metabolic alterations in the gut microbiota different from those induced by other diseases

Sergio Serrano-Villar, David Rojo, Mónica Martínez-Martínez, Simon Deusch, Jorge F Vázquez-Castellanos, Talía Sainz, Mar Vera, Santiago Moreno, Vicente Estrada, María José Gosalbes, Amparo Latorre, Abelardo Margolles, Jana Seifert, Coral Barbas, Andrés Moya, Manuel Ferrer^*^

**SUPPLEMENTARY TABLES**

Supplementary Table S1. Individual characteristics, clinical variables and HIV immune pathogenesis markers. For HIV-infected patients, the time since HIV diagnosis (years, interquartile range [IQR[) was 5.4 (2.9-9.9) for VU patients, 8.0 (4.5-13.9) for IR patients and 11.8 (5.6-20.8) for INR patients); the duration of ART regime was (years, IQR) 6.1 (4.3-11.7) for IR patients and 8.9 (4.6-12.7) for INR patients; the HIV RNA level (copies/mL, IQR) was 22198 (9955-40621) for VU patients and <20 for IR and INR patients; and the Framingham Risk Score was (%, IQR) 2.73 (2-1) for VU, 5.23 (7-2) for IR and 5.29 (6-4) for INR patients. Other innate immune activation and bacterial translocation markers for HIV infected patients are extensively described in the Table (data not available for SLE, CDADt+ patients and healthy controls). Due to the extensive size this file is submitted as separate Excel file. Abbreviations: NaN, data not available.

Supplementary Table S2. List of masses identified and quantified by metabolomic approaches using LC-MS (-/+) in the gut microbiota of the investigated VU, IR, INR, CDADt^+^, HCh and HCl individuals. For differential quantitative metabolomics, we compared the metabolomes (in triplicate) of gut bacterial cells by evaluating the peak area from the chromatographic peaks. A list of masses that were identified by LC-MS using both positive (Panel A) and negative (Panel B) polarities is presented for all individuals. Because the samples interact during the separation technique and MS, it is crucial to employ quality controls (QCs) during LC-MS to ensure analytical reproducibility. Indeed, QC samples are required throughout the analytical runs at periodic intervals during analysis to monitor variations in the signals across time and at the beginning of the sequence to stabilize the system. QC samples were prepared for LC-MS by pooling and mixing equal volumes of each sample. After gently vortexing the samples, the mixture was filtered and subsequently transferred to an analytical vial and analysed. In all cases, the mass (in Dalton [Da]), retention time (RT; as Da@RT), abundance and metabolite identity (for those for which identity was unambiguously established) in each sample are shown. Due to the extensive size this file is submitted as separate Excel file.

Supplementary Table S3. List of identified proteins and determined label-free quantification (LFQ) values. Due to the extensive size this file is submitted as separate Excel file.
